# Exploring the Role of Environmental Factors on Chromosomal Translocations Associated With Childhood Leukaemia

**DOI:** 10.1002/cam4.71713

**Published:** 2026-03-08

**Authors:** Jessica R. Saville, Lisa J. Russell, Kay Padget, Jill A. McKay

**Affiliations:** ^1^ School of Geography and Natural Sciences, Faculty of Science and Environment, Northumbria University Newcastle upon Tyne UK; ^2^ Biosciences Institute, Newcastle University Centre for Cancer, Faculty of Medical Sciences, Newcastle University Newcastle upon Tyne UK; ^3^ St. George's University, Grenada, Based at Northumbria University London UK

**Keywords:** benzene, cotinine, maternal caffeine, maternal folate, maternal smoking

## Abstract

**Introduction:**

Leukaemia is the most common cancer in children, with incidence rates increasing. Chromosomal translocations are considered one of the leukaemia‐initiating events; however, the causes of many translocations remain unknown. Epidemiological studies have identified environmental exposures associated with altered risk of childhood leukaemia; however, there is little understanding of the molecular role they play in the aetiology of leukaemia. It is plausible that they contribute to the induction of translocations.

**Methods:**

In this exploratory study, in vitro techniques were used to screen for the induction of translocations in response to environmental exposures suggested to be associated with childhood leukaemia risk that is, caffeine, benzene (smoking/air pollution), cotinine (smoking) and folate. Using physiologically relevant concentrations, NALM6 cells were exposed to each risk factor for up to 96 h. Reverse transcription PCR assays were used to detect common childhood leukaemia‐associated translocations, *TCF3::PBX1* and *RUNX1::RUNX1T1*.

**Results:**

Preliminary experiments observed *TCF3::PBX1* and *RUNX1::RUNX1T1* translocations in benzene and cotinine exposed cells, including concentrations equivalent to passive smoke exposure. *TCF3::PBX1* translocations were observed in caffeine‐exposed cells at concentrations observed during pregnancy. *TCF3::PBX1* and *RUNX1::RUNX1T1* translocations were observed in cells grown in varying folic acid concentrations including levels within the normal physiological range.

**Conclusions:**

Our preliminary data provides proof of principle to suggest that environmental factors associated with childhood leukaemia risk have the potential to induce chromosomal translocations. Whilst this study is not designed to estimate in vivo risk or translocation frequency, it has allowed us to demonstrate a biologically plausible mechanism for epidemiological associations. Understanding the risk factors contributing to leukaemia‐initiating events will be essential to refine public health policy and tailor prevention strategies.

## Graphical abstract

Our study provides preliminary proof of principle that physiologically relevant levels of caffeine, benzene, cotinine and folic acid, risk factors of childhood leukaemia, may influence chromosomal translocation induction. Understanding the environmental contribution to leukaemia‐initiating chromosomal translocations will be essential to develop preventative interventions to reduce the incidence of childhood leukaemia.

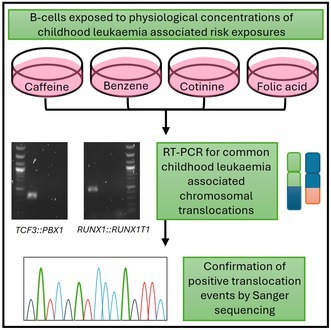

**Abbreviations**
ALL: acute lymphoblastic leukaemia
DMSO: dimethylsulfoxide
FBS: foetal bovine serum
FISH: fluorescent in situ hybridisation
P/S: penicillin/streptomycin
ROS: reactive oxygen species
RT‐PCR: reverse transcription‐PCR

## 1 Introduction

Childhood leukaemia is the most common cancer in children, accounting for nearly a third of all childhood cancers [1]. Incidence of childhood leukaemia shows a 1% increase each year [2], suggesting an environmental contribution to risk. Epidemiological studies have identified various in utero environmental exposures as potential risk factors for childhood leukaemia. Although the magnitude of the observed associations is variable and sometimes inconsistent, factors that have been implicated to increase the risk of childhood leukaemia include maternal and paternal smoking [3] and maternal caffeine intake [4, 5, 6]. Maternal supplementation with folic acid during pregnancy has been suggested to decrease risk [7, 8]. The underlying mechanisms through which these in utero exposures may infer risk are poorly understood; therefore, further understanding of the biological mechanisms through which they may contribute to childhood leukaemia development will be important to reduce incidence rates.

Using cord blood and blood from newborn heel pricks, multiple studies have identified the presence of leukaemia‐associated chromosomal translocations at birth, indicating that these events occurred in utero [9, 10]. Chromosomal translocations occur when there are two breaks on different chromosomes, which occur in spatial and temporal proximity, such that incorrect ends are joined together, which can result in fusion gene products and misregulation of genes [11]. It is plausible that some exposures suggested to influence childhood leukaemia risk may do so by contributing to the incidence of chromosomal translocations, as some exposures are known to influence DNA structure.

Benzene, a major carcinogen of cigarette smoke, causes DNA damage both in vitro and in animal models [12, 13]. It can travel across the placenta, exposing offspring in utero at concentrations equal to or higher than maternal concentrations [14], and therefore could potentially influence the developing foetal genome. Another component of cigarette smoking is nicotine, largely metabolised to cotinine, with offspring exposed in utero at levels observed in active smokers [15] and evidence of DNA damage has been observed in vitro [16]. In addition, in vitro studies have shown that caffeine exposure can impact DNA, including altering DNA repair and cell cycle checkpoints [17]. Finally, folate is key in DNA replication due to its role in one‐carbon metabolism and the synthesis of purines and pyrimidines [18] and under deficient conditions, DNA damage, including double‐strand breaks, has been observed both in vivo and in vitro [19, 20].

As DNA damage can lead to DNA double‐strand breaks [21], known to be potent inducers of chromosomal translocations [22], we hypothesised that it is plausible that environmental factors associated with increased risk of childhood leukaemia, and are known to cause DNA damage, may influence chromosomal translocation events and thus be one potential mechanism driving those associations. In a highly exploratory study, we exposed NALM6 cells, a pre‐B lymphoblastic cell line with compromised mismatch repair response, to a range of physiological levels of selected exposures to investigate the role of these exposures on the potential formation of chromosomal translocations that are commonly associated with childhood leukaemia. Given the potential to influence DNA damage, we selected benzene and cotinine as proxy exposures for smoking and caffeine and folic acid as exposures for investigation.

## 2 Methods

### 2.1 Cell Culture

NALM6 cells (DSMZ, Germany, RRID:CVCL_0092), were expanded in RPMI‐1640 (Gibco, Fisher Scientific, UK) with 10% foetal bovine serum (FBS, Gibco, Fisher Scientific, UK) and 1% penicillin/streptomycin (P/S, Gibco, Fisher Scientific, UK), incubated at 37°C and 5% CO_2_. The NALM6 cell line used in this study was authenticated by STR profiling, performed by Eurofins Genomics (Eurofins, Germany), and compared with the CLASTR 1.4.4 Cellosaurus STR Similarity Search Tool [23] (https://www.cellosaurus.org/str‐search/) with a score of 81.08% indicating a good match with NALM6. The NALM6 cell line was confirmed as mycoplasma free through routine mycoplasma testing using VenorGeM OneStep (Minerva Biolabs, Berlin, Germany) following the manufacturer's instructions. Cell counts and viability were assessed every 24 h using a haemocytometer and trypan blue staining [24]. Cells were seeded at 0.5 × 10^6^ cells/mL in RPMI‐1640, treated with benzene (Scientific Laboratory Supplies, UK), cotinine (Fisher Scientific, UK), caffeine (Scientific Laboratory Supplies, UK) or dimethylsulfoxide (DMSO, Fisher Scientific, UK), (Table S1), and harvested after 2 and 4 days. For folic acid treatment, cells were washed and seeded in folic acid free RPMI‐1640 (Gibco, Fisher Scientific, UK, supplemented with 10% FBS and 1% P/S), at 0.5 × 10^6^ cells/mL, treated with folic acid (Fisher Scientific, UK) dissolved in DMSO, (Table S1), and harvested after 4 days (suggested to deplete endogenous folate whist maintaining cell viability [25]). Three biological replicates were performed per exposure and 10–20 replicates for reproducibility experiments.

### 2.2 RT‐PCR

RNA was isolated using EZNA Total RNA Isolation Kit I (Omega Bio‐Tek, USA), treated with DNase (Primer Design, UK) and reverse transcribed using Precision nanoScript2 Reverse Transcription Kit (Primer Design, UK). PCR was performed on a 6331 Nexus Gradient MasterCycler Thermal Cycler (Eppendorf, Germany) with a master‐mix of PCRBIO 2X HS Taq Mix (PCR Biosystems, UK) and 10 pmol of primers (Eurofins, Germany). *TCF3::PBX1* primers, forward: GGCCTGCAGAGTAAGATAG, and reverse: CACGCCTTCCGCTAACAG. *RUNX1::RUNX1T1* primers, forward: GAGGGAAAAGCTTCACTCTG and reverse: GCGAACTCTTTCTCCTATC. Translocation‐positive cell lines pre‐B 697 (*TCF3::PBX1*) and Kasumi‐1 (*RUNX1::RUNX1T1*) were used as a positive control, with 5 ng/μl of cDNA. Cycling conditions included denaturation (95°C, 2 min), 40 cycles of denaturation (95°C, 15 s), annealing (60°C, 15 s), and extension (72°C, 30 s), and a final extension (72°C, 2 min). Visualisation with gel electrophoresis was performed with 1% agarose gel containing Sybr Safe (Invitrogen, USA).

Sanger sequencing was performed on positive RT‐PCR controls and a sub‐sample of translocation‐positive exposure cells to confirm alignment with expected translocations. RT‐PCR products were purified using GeneJET PCR purification/gel extraction kit (Fisher Scientific, UK) and sent to DBS Genomics (Durham University, UK) for Sanger sequencing using Applied Biosciences 3730 capillary instrument. Forward and reverse sequencing reactions used 3.2 pmol/μl primer with > 10 ng/μl DNA. Sequencing data were submitted to the Basic Local Alignment Search Tool (BLAST, National Center for Biotechnology Information) for sequence comparison.

## 3 Results

### 3.1 Exposure to Benzene, Cotinine, Caffeine and Folic Acid Induces Chromosomal Translocations in NALM6

NALM6 were incubated for 2 or 4 days with physiologically relevant concentrations of benzene, cotinine, caffeine and folic acid (Table 1). Exposure to all risk factors resulted in decreased cell viability over time (Figure S1). However, for all exposures, over 50% of cells were viable at the time of harvesting.

**TABLE 1:** Translocation events in response to environmental exposures and their corresponding physiological range as reported in literature (References in Table S1). Each box highlighted represents the number of replicates where a translocation event was observed across 3 biological experiments. For boxes in grey, no translocation events were measured.

Exposure	Conc.	Physiological range	*TCF3::PBX1* (3 replicates)		*RUNX1::RUNX1T1* (3 replicates)	
			Day 2	Day 4	Day 2	Day 4
DMSO	0.01%	Control				
Benzene	1.5 nM	Non‐smoker	1	1		
	3 nM	Smoker			1	
	6 nM	Benzene worker				
	12 nM	Heavy smoker		1		
	24 nM	Benzene worker smoker				1
	48 nM	Extreme			2	1
Cotinine	10 nM	Non‐smoker	1			
	100 nM	Second‐hand smoke	1			
	1 μM	Average smoker				
	5 μM	Very heavy smoker			1	
	10 μM	Extreme exposure				
Caffeine	2 μM	Low intake				
	10 μM	Medium intake/average 1st trimester	1			
	20 μM	Average 3rd trimester				
	40 μM	Very high 3rd trimester				
	80 μM	Extreme		1		
Folic acid	2000 μM	Normal TC media (control)				
	200 nM	Very high				
	100 nM	High				1
	50 nM	High‐end normal range				
	10 nM	Low‐end normal range		1		
	5 nM	Low				
	1 nM	Depleted		1		
	0.1 nM	Deficient				

RT‐PCR on cDNA synthesised from DMSO treated and 2000 nM folic acid treated (to mimic standard media) cells, that is, unexposed controls, resulted in no amplification of the target translocations *TCF3::PBX1* and *RUNX1::RUN1T1* (Table 1). The highest number of translocations were observed in response to benzene, *n* = 8, followed by folate depletion, *n* = 3, whilst cotinine and caffeine exposure were at similar levels, that is, *n* = 3 and *n* = 2 respectively (Table 1).

In benzene‐exposed cells, at least one translocation was observed for most concentrations tested, and an equal number of translocations were observed at 2 and 4 days (*n* = 4). There was some evidence of recurrent events that is, *TCF3::PBX1* observed in response to 1.5 nM benzene after both 2 and 4 days exposure, and two *RUNX1::RUNX1T1* events observed after 2 days exposure of 48 nm benzene and one observed after 4 days.

In cotinine‐exposed cells, a single event was observed for three of five concentrations tested. There was some evidence to suggest that different translocation events occur in response to high and low cotinine concentrations with *TCF3::PBX1* observed at the lowest concentrations of cotinine exposure that is, 10 nM and 100 nM after 2 days, and *RUNX1::RUNX1T1* occurring following exposure to a high cotinine concentration, that is, 5 μM after 2 days.

Translocations were only observed for two of five caffeine concentrations tested, with a single *TCF3::PBX1* translocation at 10 μM after 2 days, and at 80 μM after 4 days.

A single translocation event was observed for three of the seven folic acid concentrations. Of these, two *TCF3::PBX1* events were observed at folic acid concentrations that are considered physiologically low, that is, 10 nM and 1 nM. A single *RUNX1::RUNX1T1* event was observed at 100 nM folic acid, considered a high physiological concentration, although low in comparison to standard tissue culture media.

Sanger sequencing for *TCF3::PBX1* positive samples (Table 2) show identical breakpoints to those observed in the pre‐B 697 control. For *RUNX1::RUNX1T1* positive samples, one sample showed an identical breakpoint to the Kasumi‐1 control, and two further samples showed the correct genes (i.e., *RUNX1* and/or *RUNX1T1*) with sequencing noise that did not match the positive breakpoint (Table 2).

**TABLE 2:** Sanger sequencing of positive translocation events.

Sample	Sequence of translocation junction	Genes identified in BLAST
Pre‐B 697 positive cell line control	*CCCGACTCCTACAGTG*TTTTGAGTATCCGAG	*TCF3* PBX1
Benzene 1.5 nM Day 2 *(TCF3::PBX1)*	*CCCGACTCCTACAGTG*TTTTGAGTATCCGAG	*TCF3* PBX1
Benzene 1.5 nM Day 4 *(TCF3::PBX1)*	*CCCGACTCCTACAGTG*TTTTGAGTATCCGAG	*TCF3* PBX1
Cotinine 100 nM Day 2 (*TCF3::PBX1*)	*CCCGACTCCTACAGTG*TTTTGAGTATCCGAG	*TCF3* PBX1
Cotinine 10 nM Day 2 (*TCF3::PBX1*)	*CCCGACTCCTACAGTG*TTTTGAGTATCCGAG	*TCF3* PBX1
Caffeine 80 μM Day 4 *(TCF3::PBX1)*	*CCCGACTCCTACAGTG*TTTTGAGTATCCGAG	*TCF3* PBX1
Kasumi‐1 positive cell line control	*GGCCCGAGAACCTCGAAAT*CGTTACTGAGAA	*RUNX1* RUNX1T1
Benzene 48 nM Day 2 *(RUNX1::RUNX1T1)*	*CCTTGAATGGCGCCCCCTCACCA*	*RUNX1T1*
Benzene 3 nM Day 2 *(RUNX1::RUNX1T1)*	*CCCCGAGAACCCTGAAAA* aaagaggGAGAAG	*RUNX1* noise RUNX1T1
Cotinine 5 μM Day 2 (*RUNX1::RUNX1T1*)	*GGGCCCCGAGAACCTCGAAAT*CGTACTGAG	*RUNX1* RUNX1T1

### 3.2 Replicability of Translocation Events in NALM6 in Response to Benzene, Cotinine, Caffeine and Folic Acid Exposure

To understand the replicability of translocation events, experiments were repeated up to 20 times for a subset of exposures where translocation events were previously observed.

Significantly more, *TCF3::PBX1* translocations were observed in response to 80 μM caffeine for 4 days (13/20 replicates), compared with DMSO controls (2/20; Fisher's exact test, *p* =< 0.0001) (Table 3). This gives an expected translocation frequency of 65% in response to caffeine exposure, suggesting that 1–2 translocation events should be observed in every three replicates, as previously observed (Table 1). Similarly, seven *TCF3::PBX1* translocations were observed in 10 replicates for 1 nM folic acid, significantly higher than the DMSO controls (Fisher's exact test, *p* = 0.0017), giving an expected translocation frequency of 70%, suggesting two events in every three replicates, whilst only one event was observed previously (Table 1). For cells exposed to 1.5 nM benzene for 2 days, only one *TCF3::PBX1* translocation was observed across 20 replicates, which was not significant compared to DMSO controls (Fisher's exact test, *p* => 0.9999).

**TABLE 3:** Translocation events detected in response to multiple replicates. For boxes in grey no translocation events were measured.

Exposure	Concentration	*TCF3::PBX1 (p)*		*RUNX1::RUNX1T1 (p)*	
		2 day	4 day	2 day	4 day
DMSO	0.01%	2/20	2/20	0/20	0/20
Benzene	1.5 nM	1/20 (> 0.9999)		5/20 (0.047)*	
	48 nM			6/20 (0.020)*	
Cotinine	5 μM			7/20 (0.0083)*	
Caffeine	80 μM		13/20 (< 0.0001)*		
Folic acid	1 nM		7/10 (0.0017)*		

* Fisher's exact test *p* = < 0.05 between exposure and corresponding DMSO control.

A significant number of *RUNX1::RUNX1T1* events were observed for benzene exposure for 2 days, including five out of 20 replicates at 1.5 nM and six out of 20 replicates at 48 nM, compared to the DMSO controls, *n* = 0 (Table 3) (Fisher's exact test, *p* = 0.047 and *p* = 0.020 respectively). This gives an expected frequency of 25%–30% for translocation events, suggesting one event in every three replicates, which is lower than the highest number of events observed in initial experiments, *n* = 2 (Table 1). A significant number of *RUNX1::RUNX1T1* events were also observed in 5 μM cotinine for 2 days that is, 7/20 replicates (Fisher's exact test, *p* = 0.0083). This gave an expected frequency of 35%, suggesting one translocation event in every three replicates, which was observed in our initial experiment (Table 1).

## 4 Discussion

Several epidemiological studies have reported associations between parental smoking, maternal caffeine intake and folic acid supplementation and childhood leukaemia risk [3, 5, 7, 26]. As these risk exposures have previously been shown to induce DNA damage and double‐strand breaks [12, 13, 16, 17, 19, 20] we hypothesised they have the potential to induce chromosomal translocations associated with childhood leukaemia. In this preliminary study, we report for the first time that exposure to physiological levels of benzene, cotinine (as components of cigarette smoke), caffeine and folic acid in vitro shows detectable chromosomal translocations associated with childhood leukaemia via RT‐PCR. These preliminary results will require further in‐depth follow‐up studies to confirm these findings.

In initial experiments, *RUNX1::RUNX1T1* events were most frequent at high benzene concentrations, associated with levels in smokers and extreme exposure, whilst *TCF3::PBX1* events were found at lower benzene concentrations. *RUNX1::RUNX1T1* events were significantly enriched across 20 additional replicates at low and high benzene concentrations, whilst no *TCF3::PBX1* events were observed. This may suggest that translocation induction in response to benzene exposure may therefore be targeted and more likely to appear in specific genomic locations. Indeed, in support of this, the *RUNX1::RUNX1T1* translocation is observed at a higher incidence in benzene‐exposed patients compared to *de novo* and therapy related AML [27]. It is important to highlight the different cell lineages examined between these findings, which may demonstrate the effect of benzene on the genome‐specific location, regardless of specific cell type.

Benzene toxicity is generally believed to be caused by benzene metabolites; however, here we suggest that exposure to benzene itself may exert genotoxic effects since NALM6 are not considered capable of benzene metabolism [28, 29]. Further investigation is warranted into the effect of benzene, as well as its metabolites, on toxicity. Benzene may contribute to leukemogenesis through mechanisms including disruption of signalling pathways, epigenetic alterations, and oxidative stress [30]. Benzene produces ROS, shown to increase double‐strand breaks and impede DNA repair [31, 32]. Measuring ROS production and DNA double‐strand break occurrence in future work will aid mechanistic understanding of how benzene exposure may induce childhood leukaemia‐associated translocations.

Cigarette smoking has well categorised carcinogens and oxidative stress inducers, known to contribute to cancer development [33], but the impact of cotinine is less known. We observed *RUNX1::RUNX1T1* translocations at cotinine concentrations associated with very heavy smokers, whereas *TCF3::PBX1* events were observed at low cotinine concentrations associated with non‐smokers. The *RUNX1::RUNX1T1* translocation is primarily found in childhood AML, which is rarer than childhood ALL, and may require a higher level of exposure to induce translocations [34]. Cotinine exposure in neuroblastoma cells observed significant differences in DNA damage index and frequency, and found increased oxidative damage in cotinine‐exposed cells [16]. This suggests that cotinine may induce double‐strand breaks through oxidative damage, and as such could lead to translocation formation. However, further investigation would be required to assess the impact of cotinine on oxidative and DNA damage in NALM6 cells.

Studies have observed associations between increased risk of ALL and consumption of two or more cups of coffee per day, equivalent to 10 μM caffeine, at which we observed translocations in our exploratory experiments [4, 6, 35, 36]. Caffeine has been shown to be a topoisomerase inhibitor, a mechanism through which chemotherapy agents such as etoposide are known to induce double‐strand breaks and translocations [37, 38]. Furthermore, through its involvement as an adenosine receptor antagonist and phosphodiesterase inhibitor, caffeine can affect DNA repair and checkpoint pathways [39]. These mechanisms should be explored in future research.

The translocation events observed in this study support the hypothesis that folate deficient conditions may plausibly induce translocations. The folate cycle is required for nucleotide synthesis with reduced folate metabolism leading to accumulation of excess uracil, which can be incorporated into DNA leading to double‐strand breaks [18, 40, 41]. One‐carbon metabolism provides methyl donors for a range of cellular processes, including the epigenome. DNA methylation is important in regulating genes involved in DNA repair, chromosome stability, and therefore, altered methylation could contribute to translocation induction [42], which should be investigated in future studies.

A strength of this exploratory study was in screening a range of exposures associated with childhood leukaemia; however, this was limited by only measuring three replicates in one cell line. Future studies should extend to test a range of cell lines and potentially organoid model systems, with larger replicate experiments for robustness of these associations.

Studies have shown some associations between risk factors and specific translocations not measured in this study, such as *ETV6::RUNX1* with caffeine and smoking [3, 43], therefore future work should also include the development of further assays to evaluate additional childhood leukaemia‐associated translocations. Furthermore, as *RUNX1::RUNX1T1* is commonly associated with AML and myeloid progenitors rather than pre‐B cells (NALM6), future validation in myeloid cell lines or CD34^+^ progenitors is required to confirm the biological relevance of these translocations [44].

Fluorescent in situ hybridisation (FISH) is the gold standard for identifying translocated cells; however, cost and time make it unrealistic as a screening tool [45]. The RT‐PCR assays used here have a detection rate of 1% for translocated cells in line with FISH detection rates (Figure S2), and so are suitable for this screening study. However, in future experiments, using digital droplet PCR would allow quantification of the number of translocated cells within each exposure sample, which would aid detection of the frequency of events. To further understand the mechanistic pathways of translocation induction by these exposures, further experimental validation should be performed including testing of intermediate DNA damage using COMET assays, oxidative stress measurements and topoisomerase complex identification using TARDIS assays [46, 47].

This exploratory study provides proof of principle that caffeine, benzene, cotinine and folic acid may impact chromosomal translocation induction, linked to key exposures associated with childhood leukaemia risk. Few prior studies have directly investigated if exposures could influence the occurrence of translocations [48, 49, 50], with no studies focusing on the specific exposures examined here. Additionally, our study is novel in addressing exposure levels of physiological relevance. Whilst this study was not designed to estimate in vivo risk or translocation frequency, it has allowed us to demonstrate a biologically plausible mechanism for epidemiological associations linking environmental exposures with childhood leukaemia risk. Whilst further in‐depth investigation is needed, this study has contributed novel data demonstrating that exposures associated with childhood leukaemia risk have the potential to cause leukaemia‐associated chromosomal translocations. Understanding the environmental contribution to leukaemia‐initiating chromosomal translocations will be important to develop preventative interventions to reduce the incidence of childhood leukaemia.

## Author Contributions

**Jessica R. Saville:** data curation (lead), formal analysis (equal), methodology (lead), writing – original draft (equal), writing – review and editing (equal). **Lisa J. Russell:** conceptualization (equal), formal analysis (equal), supervision (supporting), writing – review and editing (equal). **Kay Padget:** conceptualization (equal), formal analysis (equal), supervision (supporting), writing – review and editing (equal). **Jill A. McKay:** conceptualization (equal), formal analysis (equal), resources (lead), supervision (lead), writing – original draft (supporting), writing – review and editing (equal).

## Funding

This work was supported by CHILDREN with CANCER UK, 19‐316. World Cancer Research Fund, IIG_FULL_2022_007. Northumbria University, RDF Studentship.

## Ethics Statement

This work has been approved by Northumbria University Ethics Committee.

## Conflicts of Interest

The authors declare no conflicts of interest.

## Supporting information


**Figure S1:** Viability of NALM6 cells. Percentage cell viability was determined by the ratio of live to dead cells using trypan blue exclusion counted every 24 h following exposure to various physiological concentrations of (A) benzene, (B) cotinine, (C) folic acid and (D) caffeine. DMSO was used as a vehicle control for benzene, cotinine and caffeine, with 2000 nM folic acid (+DMSO as a vehicle control) was used as a standard media control for folic acid. Two replicates are reported for cotinine, folic acid and caffeine, showing standard error. One replicate is reported for benzene.
**Figure S2:** Gel electrophoresis image for RT‐PCR amplification of (A) TCF3::PBX1 and (B) RUNX1::RUNX1T1. The positive cell line for each translocation was diluted with a negative cell line at different percentages before RNA extraction and reverse transcription. The final cDNA concentration used was 5 ng/μl. MWM = molecular weight marker (New England Biosciences 100 bp DNA ladder).
**Table S1:** List of concentrations used in exposure experiments and corresponding literature used to reflect physiological levels.

## Data Availability

The data that support the findings of this study are available from the corresponding author upon reasonable request.

## References

[1] UK CwC , “Childhood Cancer Facts & Figures,” (2018), https://www.childrenwithcancer.org.uk/childhood‐cancer‐info/childhood‐cancer‐facts‐figures/.

[2] WHO , “Incidence of Childhood Leukaemia, an ENHIS Fact Sheet,” (2020), https://www.euro.who.int/en/health‐topics/noncommunicable‐diseases/cancer/publications/2009/4.1‐incidence‐of‐childhood‐leukaemia,‐an‐enhis‐fact‐sheet.

[3] C. Metayer , L. Zhang , J. L. Wiemels , et al., “Tobacco Smoke Exposure and the Risk of Childhood Acute Lymphoblastic and Myeloid Leukemias by Cytogenetic Subtype,” Cancer Epidemiology, Biomarkers & Prevention 22, no. 9 (2013): 1600–1611, 10.1158/1055-9965.EPI-13-0350. PMC3769478 23853208

[4] F. Menegaux , C. Steffen , S. Bellec , et al., “Maternal Coffee and Alcohol Consumption During Pregnancy, Parental Smoking and Risk of Childhood Acute Leukaemia,” Cancer Detection and Prevention 29, no. 6 (2005): 487–493, 10.1016/j.cdp.2005.06.008. 16289502

[5] E. Milne , K. R. Greenop , E. Petridou , et al., “Maternal Consumption of Coffee and Tea During Pregnancy and Risk of Childhood ALL: A Pooled Analysis From the Childhood Leukemia International Consortium,” Cancer Causes & Control 29, no. 6 (2018): 539–550, 10.1007/s10552-018-1024-1. 29600472

[6] J. Blanco‐Lopez , I. Iguacel , S. Pisanu , et al., “Role of Maternal Diet in the Risk of Childhood Acute Leukemia: A Systematic Review and Meta‐Analysis,” International Journal of Environmental Research and Public Health 20, no. 7 (2023) 5428, 10.3390/ijerph20075428. 37048042 PMC10093835

[7] A. Amigou , J. Rudant , L. Orsi , et al., “Folic Acid Supplementation, MTHFR and MTRR Polymorphisms, and the Risk of Childhood Leukemia: The ESCALE Study (SFCE),” Cancer Causes & Control 23, no. 8 (2012): 1265–1277, 10.1007/s10552-012-0004-0. 22706675

[8] W. R. Wan Ismail , R. Abdul Rahman , N. A. A. Rahman , A. Atil , and A. M. Nawi , “The Protective Effect of Maternal Folic Acid Supplementation on Childhood Cancer: A Systematic Review and Meta‐Analysis of Case‐Control Studies,” Journal of Preventive Medicine and Public Health 52, no. 4 (Jul 2019): 205–213, 10.3961/jpmph.19.020. 31390683 PMC6686110

[9] D. Schafer , M. Olsen , D. Lahnemann , et al., “Five Percent of Healthy Newborns Have an ETV6‐RUNX1 Fusion as Revealed by DNA‐Based GIPFEL Screening,” Blood 131, no. 7 (2018): 821–826. 29311095 10.1182/blood-2017-09-808402 PMC5909885

[10] J. Zuna , J. Madzo , O. Krejci , et al., “ETV6/RUNX1 (TEL/AML1) is a Frequent Prenatal First Hit in Childhood Leukemia,” Blood 117, no. 1 (2011): 368–369. 21212293 10.1182/blood-2010-09-309070

[11] J. Zheng , “Oncogenic Chromosomal Translocations and Human Cancer (Review),” Oncology Reports 30, no. 5 (2013): 2011–2019, 10.3892/or.2013.2677. 23970180

[12] A. Lau , C. L. Belanger , and L. M. Winn , “In Utero and Acute Exposure to Benzene: Investigation of DNA Double‐Strand Breaks and DNA Recombination in Mice,” Mutation Research 676, no. 1–2 (2009): 74–82, 10.1016/j.mrgentox.2009.04.001. 19486867

[13] D. Peng , W. Jiaxing , H. Chunhui , P. Weiyi , and W. Xiaomin , “Study on the Cytogenetic Changes Induced by Benzene and Hydroquinone in Human Lymphocytes,” Human & Experimental Toxicology 31, no. 4 (2012): 322–335, 10.1177/0960327111433900. 22297702

[14] B. J. Dowty , J. L. Laseter , and J. Storer , “The Transplacental Migration and Accumulation in Blood of Volatile Organic Constituents,” Pediatric Research 10, no. 7 (1976): 696–701, 10.1203/00006450-197607000-00013. 934736

[15] L. G. Spector , S. E. Murphy , K. M. Wickham , B. Lindgren , and A. M. Joseph , “Prenatal Tobacco Exposure and Cotinine in Newborn Dried Blood Spots,” Pediatrics 133, no. 6 (2014): e1632–e1638, 10.1542/peds.2013-3118. 24819573 PMC4035592

[16] D. Dalberto , C. C. Nicolau , A. L. H. Garcia , A. P. Nordin , I. Grivicich , and J. D. Silva , “Cytotoxic and Genotoxic Evaluation of Cotinine Using Human Neuroblastoma Cells (SH‐SY5Y),” Genetics and Molecular Biology 43, no. 2 (2020): e20190123, 10.1590/1678-4685-gmb-2019-0123. 32478795 PMC7271658

[17] M. Tsabar , V. V. Eapen , J. M. Mason , et al., “Caffeine Impairs Resection During DNA Break Repair by Reducing the Levels of Nucleases Sae2 and Dna2,” Nucleic Acids Research 43, no. 14 (2015): 6889–6901, 10.1093/nar/gkv520. 26019182 PMC4538808

[18] C. E. Clare , A. H. Brassington , W. Y. Kwong , and K. D. Sinclair , “One‐Carbon Metabolism: Linking Nutritional Biochemistry to Epigenetic Programming of Long‐Term Development,” Annual Review of Animal Biosciences 7 (2019): 263–287, 10.1146/annurev-animal-020518-115206. 30412672

[19] G. Bistulfi , E. Vandette , S. Matsui , and D. J. Smiraglia , “Mild Folate Deficiency Induces Genetic and Epigenetic Instability and Phenotype Changes in Prostate Cancer Cells,” BMC Biology 8, no. 1 (2010): 6, 10.1186/1741-7007-8-6. 20092614 PMC2845099

[20] N. Lamm , K. Maoz , A. C. Bester , et al., “Folate Levels Modulate Oncogene‐Induced Replication Stress and Tumorigenicity,” EMBO Molecular Medicine 7, no. 9 (2015): 1138–1152, 10.15252/emmm.201404824. 26197802 PMC4568948

[21] E. Mladenov and G. Iliakis , “Induction and Repair of DNA Double Strand Breaks: The Increasing Spectrum of Non‐Homologous End Joining Pathways,” Mutation Research, Fundamental and Molecular Mechanisms of Mutagenesis 711, no. 1 (2011): 61–72, 10.1016/j.mrfmmm.2011.02.005. 21329706

[22] L. F. Povirk , “Biochemical Mechanisms of Chromosomal Translocations Resulting From DNA Double‐Strand Breaks,” DNA Repair (Amst) 5, no. 9–10 (2006): 1199–1212, 10.1016/j.dnarep.2006.05.016. 16822725

[23] T. Robin , A. Capes‐Davis , and A. Bairoch , “CLASTR: The Cellosaurus STR Similarity Search Tool—A Precious Help for Cell Line Authentication,” International Journal of Cancer 146, no. 5 (2020): 1299–1306, 10.1002/ijc.32639. 31444973

[24] W. Strober , “Monitoring Cell Growth,” Current Protocols in Immunology (2001), 10.1002/0471142735.ima03as21. 18432653

[25] H. Matsue , K. G. Rothberg , A. Takashima , B. A. Kamen , R. G. Anderson , and S. W. Lacey , “Folate Receptor Allows Cells to Grow in Low Concentrations of 5‐Methyltetrahydrofolate,” Proceedings of the National Academy of Sciences of the United States of America 89, no. 13 (1992): 6006–6009, 10.1073/pnas.89.13.6006. 1631087 PMC402127

[26] E. M. John , D. A. Savitz , and D. P. Sandler , “Prenatal Exposure to Parents' Smoking and Childhood Cancer,” American Journal of Epidemiology 133, no. 2 (1991): 123–132, 10.1093/oxfordjournals.aje.a115851. 1822074

[27] R. D. Irons , Y. Chen , X. Wang , J. Ryder , and P. J. Kerzic , “Acute Myeloid Leukemia Following Exposure to Benzene More Closely Resembles de Novo Than Therapy Related‐Disease,” Genes, Chromosomes & Cancer 52, no. 10 (2013): 887–894, 10.1002/gcc.22084. 23840003

[28] Atlas THP , “CYP2E1,” (2024), https://www.proteinatlas.org/ENSG00000130649‐CYP2E1/cell+line#leukemia.

[29] M. Uhlén , L. Fagerberg , B. M. Hallström , et al., “Tissue‐Based Map of the Human Proteome,” Science 347, no. 6220 (2015): 1260419, 10.1126/science.1260419. 25613900

[30] C. M. McHale , L. Zhang , and M. T. Smith , “Current Understanding of the Mechanism of Benzene‐Induced Leukemia in Humans: Implications for Risk Assessment,” Carcinogenesis 33, no. 2 (2012): 240–252, 10.1093/carcin/bgr297. 22166497 PMC3271273

[31] N. R. Pannunzio and M. R. Lieber , “Concept of DNA Lesion Longevity and Chromosomal Translocations,” Trends in Biochemical Sciences 43, no. 7 (2018): 490–498, 10.1016/j.tibs.2018.04.004. 29735400 PMC6014902

[32] A. Sallmyr , J. Fan , and F. V. Rassool , “Genomic Instability in Myeloid Malignancies: Increased Reactive Oxygen Species (ROS), DNA Double Strand Breaks (DSBs) and Error‐Prone Repair,” Cancer Letters 270, no. 1 (2008): 1–9, 10.1016/j.canlet.2008.03.036. 18467025

[33] A. W. Caliri , S. Tommasi , and A. Besaratinia , “Relationships Among Smoking, Oxidative Stress, Inflammation, Macromolecular Damage, and Cancer,” Mutation Research, Reviews in Mutation Research 787 (2021): 108365, 10.1016/j.mrrev.2021.108365. 34083039 PMC8287787

[34] M. F. Greaves and J. Wiemels , “Origins of Chromosome Translocations in Childhood Leukaemia,” Nature Reviews. Cancer 3, no. 9 (2003): 639–649, 10.1038/nrc1164. 12951583

[35] A. Bonaventure , J. Rudant , S. Goujon‐Bellec , et al., “Childhood Acute Leukemia, Maternal Beverage Intake During Pregnancy, and Metabolic Polymorphisms,” Cancer Causes & Control 24, no. 4 (2013): 783–793, 10.1007/s10552-013-0161-9. 23404349

[36] F. Menegaux , M. Ripert , D. Hémon , and J. Clavel , “Maternal Alcohol and Coffee Drinking, Parental Smoking and Childhood Leukaemia: A French Population‐Based Case‐Control Study,” Paediatric and Perinatal Epidemiology 21, no. 4 (2007): 293–299, 10.1111/j.1365-3016.2007.00824.x. 17564585

[37] I. G. Cowell , Z. Sondka , K. Smith , et al., “Model for MLL Translocations in Therapy‐Related Leukemia Involving Topoisomerase IIbeta‐Mediated DNA Strand Breaks and Gene Proximity,” Proceedings of the National Academy of Sciences of the United States of America 109, no. 23 (2012): 8989–8994, 10.1073/pnas.1204406109. 22615413 PMC3384169

[38] C. G. Shin , J. M. Strayer , M. A. Wani , and R. M. Snapka , “Rapid Evaluation of Topoisomerase Inhibitors: Caffeine Inhibition of Topoisomerases in Vivo,” Teratogenesis, Carcinogenesis, and Mutagenesis 10, no. 1 (1990): 41–52. 1971968 10.1002/tcm.1770100106

[39] M. Porta , J. Vioque , D. Ayude , et al., “Coffee Drinking the Rationale for Treating It as a Potential Effect Modifier of Carcinogenic Exposures,” European Journal of Epidemiology 18, no. 4 (2003): 289–298. 12803368 10.1023/a:1023700216945

[40] B. C. Blount , M. M. Mack , C. M. Wehr , et al., “Folate Deficiency Causes Uracil Misincorporation Into Human DNA and Chromosome Breakage: Implications for Cancer and Neuronal Damage,” Proceedings of National Academy of Sciences 94, no. 7 (1997): 3290–3295. 10.1073/pnas.94.7.3290 PMC20362 9096386

[41] S. J. Duthie and A. R. Hawdon , “DNA Instability (Strand Breakage, Uracil Misincorporation, and Defective Repair) is Increased by Folic Acid Depletion in Human Lymphocytes in Vitro,” FASEB Journal 12 (1998): 1491–1497. 9806758

[42] H. Meng , Y. Cao , J. Qin , et al., “DNA Methylation, Its Mediators and Genome Integrity,” International Journal of Biological Sciences 11, no. 5 (2015): 604–617, 10.7150/ijbs.11218. 25892967 PMC4400391

[43] E. Milne , J. A. Royle , L. C. Bennett , et al., “Maternal Consumption of Coffee and Tea During Pregnancy and Risk of Childhood ALL: Results From an Australian Case‐Control Study,” Cancer Causes & Control 22, no. 2 (2011): 207–218, 10.1007/s10552-010-9688-1. 21113653

[44] K. N. Manola , “Cytogenetics of Pediatric Acute Myeloid Leukemia,” European Journal of Haematology 83, no. 5 (2009): 391–405, 10.1111/j.1600-0609.2009.01308.x. 19563518

[45] L. Hu , K. Ru , L. Zhang , et al., “Fluorescence in Situ Hybridization (FISH): An Increasingly Demanded Tool for Biomarker Research and Personalized Medicine,” Biomarker Research 2, no. 1 (2014): 3, 10.1186/2050-7771-2-3. 24499728 PMC3917523

[46] I. G. Cowell and C. A. Austin , “Visualization and Quantification of Topoisomerase‐DNA Covalent Complexes Using the Trapped in Agarose Immunostaining (TARDIS) Assay,” Methods in Molecular Biology 1703 (2018): 301–316, 10.1007/978-1-4939-7459-7_21. 29177750

[47] M. Kuchařová , M. Hronek , K. Rybáková , et al., “Comet Assay and Its Use for Evaluating Oxidative DNA Damage in Some Pathological States,” Physiological Research 68, no. 1 (2019): 1–15, 10.33549/physiolres.933901. 30433808

[48] B. Bariar , C. G. Vestal , B. Deem , et al., “Bioflavonoids Promote Stable Translocations Between MLL‐AF9 Breakpoint Cluster Regions Independent of Normal Chromosomal Context: Model System to Screen Environmental Risks,” Environmental and Molecular Mutagenesis 60, no. 2 (2019): 154–167, 10.1002/em.22245. 30387535 PMC6363851

[49] S. van Barjesteh Waalwijk Doorn‐Khosroi , J. Janssen , L. M. Maas , R. W. Godschalk , J. G. Nijhuis , and F. J. van Schooten , “Dietary Flavonoids Induce MLL Translocations in Primary Human CD34+ Cells,” Carcinogenesis 28, no. 8 (2007): 1703–1709, 10.1093/carcin/bgm102. 17468513

[50] R. Strick , P. L. Strissel , S. Borgers , S. L. Smith , and J. D. Rowley , “Dietary Bioflavonoids Induce Cleavage in the MLL Gene and May Contribute to Infant Leukemia,” Proceedings of the National Academy of Sciences of the United States of America 97, no. 9 (2000): 4790–4795, 10.1073/pnas.070061297. 10758153 PMC18311

