# Supplementary material for: Exploring the Role of Environmental Factors on Chromosomal Translocations Associated With Childhood Leukaemia
Source: Cancer Med. 2026 Mar 8;15(3):e71713. doi: 10.1002/cam4.71713 (PMC12967601; doi:10.1002/cam4.71713)
Supplement: Supplementary file 1 — Figure S1: Viability of NALM6 cells. Percentage cell viability was determined by the ratio of live to dead cells using trypan blue exclusion counted every 24 h following exposure to various physiological concentrations of (A) benzene, (B) cotinine, (C) folic acid and (D) caffeine. DMSO was used as a vehicle control for benzene, cotinine and caffeine, with 2000 nM folic acid (+DMSO as a vehicle control) was used as a standard media control for folic acid. Two replicates are reported for cotinine, folic acid and caffeine, showing standard error. One replicate is reported for benzene. Figure S2: Gel electrophoresis image for RT‐PCR amplification of (A) TCF3::PBX1 and (B) RUNX1::RUNX1T1. The positive cell line for each translocation was diluted with a negative cell line at different percentages before RNA extraction and reverse transcription. The final cDNA concentration used was 5 ng/μl. MWM = molecular weight marker (New England Biosciences 100 bp DNA ladder). Table S1: List of concentrations used in exposure experiments and corresponding literature used to reflect physiological levels. [file CAM4-15-e71713-s001.docx]

**Supplementary Materials**


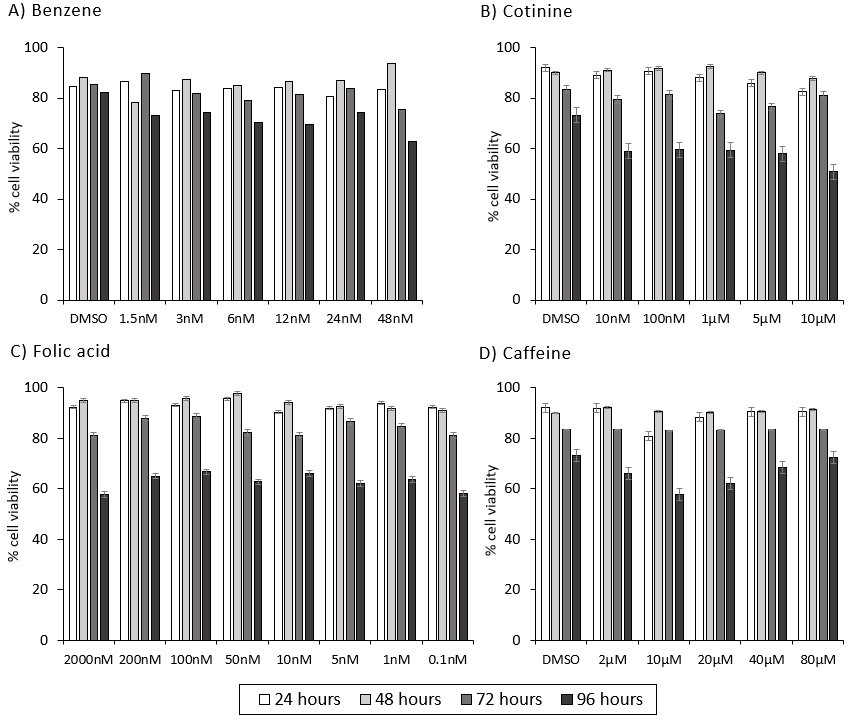


Figure S1. Viability of NALM6 cells. Percentage cell viability was determined by the ratio of live to dead cells using trypan blue exclusion counted every 24 hours following exposure to various physiological concentrations of A) benzene, B) cotinine, C) folic acid, and D) caffeine. DMSO was used as a vehicle control for benzene, cotinine and caffeine, with 2000nM folic acid (+DMSO as a vehicle control) was used as a standard media control for folic acid. Two replicates are reported for cotinine, folic acid and caffeine, showing standard error. One replicate is reported for benzene.


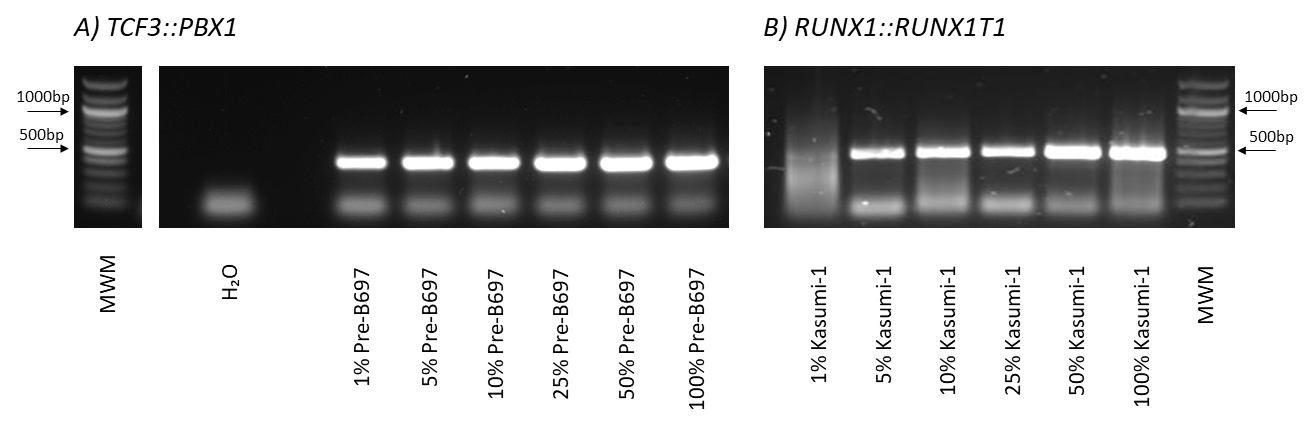


Figure S2. Gel electrophoresis image for RT-PCR amplification of A) TCF3::PBX1 and B) RUNX1::RUNX1T1. The positive cell line for each translocation was diluted with a negative cell line at different percentages before RNA extraction and reverse transcription. The final cDNA concentration used was 5ng/µl. MWM = molecular weight marker (New England Biosciences 100 bp DNA Ladder).

Table S1. List of concentrations used in exposure experiments and corresponding literature used to reflect physiological levels.

| Exposure | Conc. | Physiological range | Reference used |
| --- | --- | --- | --- |
| DMSO | 0.01% | Control | N/A |
| Benzene | 1.5nM | Non-smoker​ | ^1^ |
|  | 3nM | Smoker​ | ^1^ |
|  | 6nM | Benzene worker​ | ^1^ |
|  | 12nM | Heavy smoker​ | ^1^ |
|  | 24nM | Benzene worker smoker​ | ^1^ |
|  | 48nM | Extreme​ | N/A |
| Cotinine | 10nM | Non-smoker​ | ^2^ |
|  | 100nM | Second-hand smoke​ | ^2^ |
|  | 1µM | Average smoker​ | ^3^ |
|  | 5µM | Very heavy smoker​ | N/A |
|  | 10µM | Extreme exposure​ | N/A |
| Caffeine | 2µM | Low intake​ | ^4^ |
|  | 10µM | Medium intake/ average 1st trimester​ | ^4, 5^ |
|  | 20µM | Average 3rd trimester​ | ^5^ |
|  | 40µM | Very high 3rd trimester​ | ^5^ |
|  | 80µM | Extreme​ | N/A |
| Folic acid | 2000µM | Normal TC media​ (Control) | N/A |
|  | 200nM | Very high​ | ^6, 7^ |
|  | 100nM | High​ | ^6, 7^ |
|  | 50nM | High end normal range​ | ^8-11^ |
|  | 10nM | Low end normal range​ | ^8-11^ |
|  | 5nM | Low​ | ^8^ |
|  | 1nM | Depleted​ | ^8^ |
|  | 0.1nM | Deficient​ | ^8^ |
| 1. Brugnone F, Perbellini L, Romeo L, et al. Benzene in environmental air and human blood *Int Arch Occup Environ Health*. 1998;71(8):554-559.  2. Kim S. Overview of Cotinine Cutoff Values for Smoking Status Classification. *Int J Environ Res Public Health*. Dec 14 2016;13(12)doi:10.3390/ijerph13121236  3. Leenders M, Chuang SC, Dahm CC, et al. Plasma cotinine levels and pancreatic cancer in the EPIC cohort study. *Int J Cancer*. Aug 15 2012;131(4):997-1002. doi:10.1002/ijc.26452  4. Klebanoff MA, Levine RJ, Dersimonian R, Clemens JD, Wilkins DG. Serum caffeine and paraxanthine as markers for reported caffeine intake in pregnancy. *Ann Epidemiol*. Feb 1998;8(2):107-11. doi:10.1016/s1047-2797(97)00125-7  5. Yu T, Campbell SC, Stockmann C, et al. Pregnancy-induced changes in the pharmacokinetics of caffeine and its metabolites. *J Clin Pharmacol*. May 2016;56(5):590-6. doi:10.1002/jcph.632  6. Bistulfi G, Vandette E, Matsui S, Smiraglia DJ. Mild folate deficiency induces genetic and epigenetic instability and phenotype changes in prostate cancer cells. *BMC Biol*. Jan 21 2010;8:6. doi:10.1186/1741-7007-8-6  7. Tomaszewski JJ, Cummings JL, Parwani AV, et al. Increased cancer cell proliferation in prostate cancer patients with high levels of serum folate. *Prostate*. Sep 2011;71(12):1287-93. doi:10.1002/pros.21346  8. WHO. Serum and red blood cell folate concentrations for assessing folate status in populations. Accessed 23/2/22, <http://apps.who.int/iris/bitstream/10665/162114/1/WHO_NMH_NHD_EPG_15.01.pdf?ua=1>  9. Wang X, Thomas P, Xue J, Fenech M. Folate deficiency induces aneuploidy in human lymphocytes in vitro-evidence using cytokinesis-blocked cells and probes specific for chromosomes 17 and 21. *Mutat Res*. Jul 13 2004;551(1-2):167-80. doi:10.1016/j.mrfmmm.2004.03.008  10. Courtemanche C, Elson-Schwab I, Mashiyama ST, Kerry N, Ames BN. Folate deficiency inhibits the proliferation of primary human CD8+ T lymphocytes in vitro. *J Immunol*. Sep 1 2004;173(5):3186-92. doi:10.4049/jimmunol.173.5.3186  11. Fenech M, Rinaldi J. A comparison of lymphocyte micronuclei and plasma micronutrients in vegetarians and non-vegetarians. *Carcinogenesis (New York)*. 1995;16(2):223-230. doi:10.1093/carcin/16.2.223 | | | |
